# Detection and Risk Prediction of Diabetes Related Kidney Disease Using Retinal Images: A Systematic Review and Meta‐Analysis

**DOI:** 10.1111/1753-0407.70271

**Published:** 2026-10-19

**Authors:** Samuel Ato Gyasi Otabir, Teketo Kassaw Tegegne, Paul Nderitu, Jiangtao Wang, Jonathan E. Shaw, David Owens, Rebecca Thomas, Robyn Jennifer Tapp, Ralph Maddison

**Affiliations:** ^1^ Centre for Intelligent Healthcare Coventry University Coventry UK; ^2^ Institute for Physical Activity and Nutrition, School of Exercise and Nutrition Sciences Deakin University Melbourne Victoria Australia; ^3^ King's Ophthalmology Research Unit (KORU), Section of Ophthalmology, Faculty of Life Sciences and Medicine King's College London London UK; ^4^ School of Computing, Engineering & Digital Technologies Teesside University Middlesbrough UK; ^5^ Baker Heart and Diabetes Institute Melbourne Victoria Australia; ^6^ Diabetes Research Group, Medical School Swansea University Swansea UK; ^7^ Melbourne School of Population and Global Health University of Melbourne Melbourne Victoria Australia

**Keywords:** artificial intelligence, deep learning, detection, diabetes kidney disease, fundus photographs, prediction, retinal images

## Abstract

**Aim:**

This study aimed to determine the effectiveness of deep learning models for detecting and predicting diabetes related kidney disease (DRKD) using two‐dimensional retinal images.

**Methods:**

A systematic review was conducted across eight databases from inception to July 14, 2026. Eligible cross‐sectional and longitudinal studies assessed deep learning models for DRKD detection or prediction using retinal images alone, clinical data alone, or hybrid models. Data on study design, model development, validation, and performance metrics were extracted, with study quality appraised using QUADAS‐2 and post hoc, PROBAST+AI. Results were synthesized narratively, with random effects meta analysis for hybrid models.

**Results:**

Of 367 studies, six met inclusion criteria. Three cross‐sectional studies of hybrid models contributed to the meta‐analysis for DRKD detection, yielding pooled AUC 0.88, sensitivity 0.80, and specificity 0.81, though heterogeneity was high (I2 93.7%–99.8%). Three further studies were synthesized narratively rather than pooled: two cross‐sectional studies used more severe or narrower CKD case definitions (AUC 0.86 and 0.96 respectively), and only one longitudinal study predicted the 5‐year risk of DRKD (AUC 0.81, sensitivity 0.70, and specificity 0.77). External validation showed hybrid models consistently outperformed retinal‐only (ΔAUC 0.03–0.11), but their advantage over clinical‐only models was inconsistent.

**Conclusion:**

Deep learning models may be useful for detecting and predicting DRKD from retinal images, with or without clinical data. However, the small number of heterogenous studies restricts generalizability. Future work should prioritize robust model development, and validation using large, diverse populations.

## Highlights

This review demonstrates that hybrid deep learning models integrating retinal images with clinical data achieved a slightly better performance (pooled AUC 0.88) in detecting and predicting DRKD compared with retinal only or clinical data only models. These findings suggest that deep learning models applied to routine retinal imaging may enable earlier identification of kidney complications in type 2 diabetes, though larger, standardized validation studies are required before clinical implementation.

## Graphical abstract

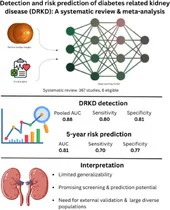

## 1 Introduction

Diabetes related kidney disease (DRKD) is a form of chronic kidney disease that develops as a complication of diabetes, and is the leading cause of end‐stage kidney disease [1]. Globally, it is estimated that almost 50% of people living with type 2 diabetes (T2D) develop DRKD [2] and the associated annual economic burden is vast [3]. DRKD is characterized by excess albumin loss in urine, structural changes in the kidneys, and a reduction in the glomerular filtration rate (GFR), progressing through five Stages [4, 5]. DRKD is asymptomatic in its early stages, and symptoms only become noticeable in the late stages [3]. The likelihood of cardiovascular co‐morbidities is increased as DRKD progresses [6]. The need for early detection of DRKD is of great importance to identify those who may benefit from early tailored intervention programs, reducing the economic and personal burden.

Current screening for DRKD entails measurement of estimated glomerular filtration rate (eGFR), typically estimated from serum creatinine, and urine albumin‐creatinine ratio (UACR), calculated from urine albumin and creatinine concentrations [7]. While these current screening techniques are generally well‐adopted, they do not provide information on the current state of the vascular beds in the kidney. The vascular beds of the kidney exhibit unique structural features that are good biomarkers of kidney function [8] and changes in the vascular beds pre‐date changes in eGFR and UACR. Moreover, the kidney and eye share similar microvasculature characteristics [9] and retinal microvascular features have been associated with kidney disease in both the general population and people living with diabetes [10, 11, 12]. Changes in retinal features such as narrower arterioles and wider venules have been associated with an increased risk of DRKD [10, 11]. This suggests that retinal images may be a useful novel biomarker for the earlier detection and prediction of DRKD using deep learning models.

Deep learning models are a subset of machine learning that use artificial neural networks with multiple layers to model and solve complex tasks [13]. These models are capable of learning useful representations of various high‐dimensional data, including medical images [14], with previous studies showing that deep learning models can detect complications such as diabetic retinopathy [15] and predict cardiovascular risk factors [16] from retinal images. A systematic review and meta‐analysis of seven studies on the diagnostic test accuracy of deep learning in screening for referable diabetic retinopathy reported a pooled sensitivity and specificity of 0.91 (95% CI: 0.79–1.00) and 0.94 (95% CI: 0.91–0.97), respectively [17]. Consistent with this, a systematic review and meta‐analysis of 4 studies on the use of deep learning for the detection of chronic kidney disease in the general population reported a pooled sensitivity and specificity of 0.88 and 0.63, respectively [18].

With regard to DRKD, to date, only one systematic review and meta‐analysis of 26 studies has attempted to assess the performance of machine learning models in predicting the condition [19]. In that review, pooled AUCs of 0.84 (95% CI: 0.79–0.89) and 0.83 (95% CI: 0.79–0.89) were reported in the internal and external validation sets, respectively [19]. However, the deep learning meta‐analysis from the review included a wide mix of data types, such as renal pathology images, renal ultrasounds, and color fundus photographs, which introduced considerable methodological heterogeneity [19]. In addition, the definition and methods used to determine DRKD varied widely across studies, representing a critical limitation that may have impacted pooled performance estimates. Thus, to date, no systematic review and meta‐analysis has specifically determined the effectiveness of deep learning in detecting and predicting DRKD primarily using retinal images, which are non‐invasive, easily accessible, and part of regular diabetic retinopathy screening. This study aimed to review and analyze the existing deep learning approaches that have been used for DRKD detection and prediction in this context.

## 2 Methods

We conducted a systematic review and meta‐analysis, which is reported according to the Preferred Reporting Items for Systematic Reviews and Meta‐Analyses (PRISMA) checklist [20]. The study protocol was registered on the International Prospective Register of Systematic Reviews (PROSPERO), ID CRD42024508334.

### 2.1 Search Strategy

A total of eight databases were searched: OVID Medline, Embase, CINAHL, Cochrane, IEEE Xplore, Web of Science, Scopus, and PsycINFO from inception to July 14, 2026. The search terms combined three key subject areas: retinal image, deep learning, and diabetic kidney disease (see Appendix S1). Manual searches of systematic reviews, meta‐analyses, and reference lists were also conducted. Search results were exported to Covidence for duplicate removal, screening, and data extraction.

### 2.2 Eligibility Criteria

The inclusion criteria included studies assessing deep learning models for detecting or predicting DRKD using a measure of eGFR or UACR. In this review, detection (as reported in the cross‐sectional studies) was defined as the identification of the presence of DRKD at the current time, while prediction (as reported in the longitudinal study) was defined as either progression from no DRKD to any stage of DRKD or progression of existing DRKD to later stages. We accepted predictions over any time horizon. Included studies had to be written in English, conducted on humans, and presented original research; conference papers were also considered eligible. We excluded studies that did not incorporate retinal images, focused on the general population, or had no subgroup analysis for people living with diabetes. In addition, we excluded studies that first extracted retinal features (e.g., vessel caliber, tortuosity, or lesion measurements) using manual methods or separate image‐analysis software before applying a prediction model rather than using end‐to‐end deep learning directly on raw retinal images. Animal studies were also excluded. The meta‐analysis specifically comprised studies that detected or predicted DRKD from retinal images with clinical data.

### 2.3 Screening and Data Extraction

Two independent reviewers (S.A.G.O. and T.K.T.) conducted title and abstract screening as well as full‐text review. Disagreements were resolved by a third reviewer (R.M.). Data extraction was conducted by one reviewer (S.A.G.O.) using Covidence and double checked by two reviewers (R.M. and T.K.T.). Extracted data included the country of study, aim, design, population description, inclusion and exclusion criteria, total participants, and population characteristics such as age, sex, ethnicity, smoking status, alcohol consumption status, height, weight, body mass index levels (BMI, kg/m^2^), systolic blood pressure (mmHg), diastolic blood pressure (mmHg), diabetes duration, history of hypertension, cardiovascular, and cerebrovascular disease, anti‐hyperglycaemic medication status, insulin use, glycated hemoglobin (HbA_1c_, %) levels, UACR (mg/g), and eGFR (ml/min/1.73m^2^). Measurement results used in assessing diagnostic accuracy or reliability of deep learning models in internal and external validation datasets were also included. Extracted data were then exported and stored in Excel.

### 2.4 Quality Assessment

One reviewer performed the quality assessment (S.A.G.O.), which was then confirmed by two reviewers (R.M. and T.K.T.). Quality assessment was performed using the Quality Assessment of Diagnostic Accuracies Studies 2 (QUADAS‐2). This assessment tool, designed for diagnostic accuracy studies, systematically evaluated the risk of bias in four domains, including patient selection, index test, reference test, and flow and timing, and concerns of the applicability of the studies regarding the review questions in all the domains except for flow and timing. While QUADAS‐2 does not capture AI‐specific methodological risks such as data partitioning, overfitting, and data leakage, it remained the most appropriate tool available at the time of protocol registration. A supplementary PROBAST+AI assessment was subsequently conducted for each included study and is provided in Appendix S3.

### 2.5 Data Analysis

A random‐effects meta‐analysis was performed to pool the performance of the deep learning models for predicting DRKD from retinal images. The restricted maximum likelihood estimator was used to calculate the heterogeneity variance estimator tau squared $\tau^{2}$. Between‐study heterogeneity was quantified using the *I* ^2^ statistic, which indicates the percentage of the variability in model performance that is due to between‐study heterogeneity rather than sampling error. The *I* ^2^ statistic was categorized into three groups: low (< 50%), moderate (50%–75%) and high (> 75%) heterogeneity. A 95% prediction interval was calculated to estimate the range in which the true effect size of a new study is expected to fall, accounting for heterogeneity across studies. All statistical analyses were performed using R.

## 3 Results

### 3.1 Study Characteristics

A total of 577 records were identified in the initial search, and 210 duplicates were removed, resulting in 367 records selected for screening with 344 records excluded after reviewing titles and abstracts. The remaining 23 articles underwent full‐text screening, and 17 studies were excluded. We also searched reference lists and found no additional eligible studies; thus six studies [21, 22, 23, 24, 25, 26] were included in this review (Figure 1). Tables 1 and 2 present the characteristics of the included studies; all participants were adults and study sample sizes ranged from 225 to 121 578 participants (development cohorts). The populations were ethnically diverse, with studies including cohorts from Singapore, Australia, Northern Ireland, and South Korea. Definitions of DRKD varied across studies. The most common approach, used by three studies [21, 22, 23], applied an eGFR < 60 mL/min/1.73m^2^ threshold. One of these [23], additionally accepted UACR ≥ 30 mg/g as an alternative qualifying criterion. One study [26] used a narrower, more severe definition, restricted to eGFR < 30 mL/min/1.73m^2^. One study [24] used a different narrower definition, restricted to CKD Stage 3 only (eGFR 30–59 mL/min/1.73m^2^), narrower than the threshold used elsewhere in this review. One study [25], using a longitudinal risk prediction design, defined incident DRKD via ICD‐9/ICD‐10 diagnostic codes and/or eGFR < 60 mL/min/1.73m^2^ at follow‐up.

**FIGURE 1:**
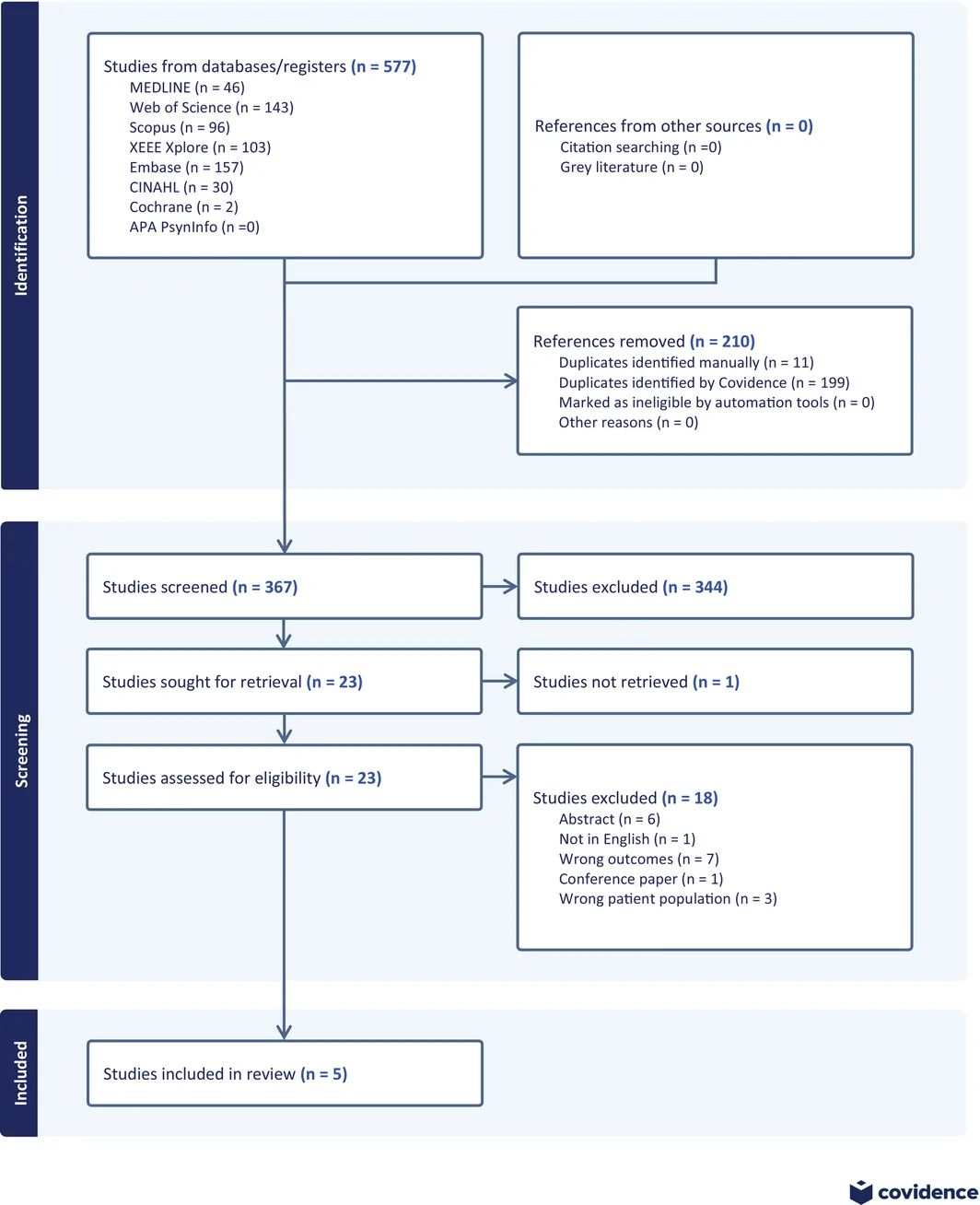
PRISMA flowchart.

**TABLE 1:** Characteristics of included studies.

No.	Title	Author and publication year	Study design and characteristics	AI architecture	Input	Retinal image capture	Reference standard	Definition of reference standard
1	Deep learning algorithms to detect diabetic kidney disease from retinal photographs in multiethnic populations with diabetes	Betzler (2023) [21]	Cross‐sectional involving people with T2D	ResNet18	Retinal images and risk factors: age, sex, ethnicity, duration of diabetes, HbA1_c_, and systolic blood pressure	Two retinal photographs (optic disc‐centered and macula‐centered) were taken from each eye using a Topcon TRC‐NW200 (Japan) digital retinal camera after pupil dilation.	eGFR	eGFR < 60 mL/min/1.73 m2 on ≥ 2 consecutive visits between 3 months to 2 years apart
2	Diagnosis of chronic kidney disease using retinal imaging and urine dipstick data: multimodal deep learning approach	Bhak (2025) [22]	Cross‐sectional involving people with T2D	XGBoost	Retinal images, risk factors (age and sex), and 5 urine dipstick measurements: glucose, specific gravity, age, pH, and sex.	Macular‐centered retinal images were taken using either TRC‐NW8, Topcon; nonmyd 7, Kowa or the VX‐10i (Kowa) with or without pupil dilation.	eGFR	eGFR < 60 mL/min/1.73 m^2^
3	Non‐invasive biopsy diagnosis of diabetic kidney disease via deep learning applied to retinal images: a population‐based study	Meng (2025) [23]	Cross‐sectional involving people with T2D	ResNet‐50 (weakly supervised momentum contrastive pretraining)	Retinal images and risk factors: age, sex, BMI, current smoking status, duration of diabetes, systolic and diastolic blood pressure, HbA1c, and diabetic retinopathy status	Not reported.	UACR & eGFR	ACR ≥ 30 mg/g or eGFR < 60 mL/min/1.73 m^2^ (eGFR < 60 mL/min/1.73 m^2^ alone in cohorts where ACR was unavailable)
4	Development and validation of a novel deep learning‐based model for detection of diabetic kidney disease from retinal imaging using a weighted loss method	Prayitnaningsih (2026) [24]	Cross‐sectional involving people with T2D	EfficientNet‐B2 (weighted cross‐entropy loss)	Retinal images only (no clinical metadata fused into the model)	Images captured using a Topcon TRC‐NW400 fundus camera by a single ophthalmologist	eGFR	eGFR 30–59 mL/min/1.73 m^2^ sustained for at least 3 months, confirmed by ≥ 2 eGFR measurements ≥ 3 months apart (CKD Stage 3)
5	A multimodal predictive model for chronic kidney disease and its association with vascular complications in patients with type 2 diabetes: Model development and validation study in South Korea and the U.K.	Cho (2025) [25]	Longitudinal risk prediction design involving people with T2D	Ensemble of VGG16 (CNN, retinal images) and a deep neural network (routine clinical metadata)	Retinal images and clinical metadata	Not reported	ICD‐9/ICD‐10 codes and/or eGFR	Incident DRKD defined via ICD‐9/ICD‐10 codes and/or eGFR < 60 mL/min/1.73m^2^ at follow‐up
6	Prediction of advanced chronic kidney disease through retinal fundus images by deep learning	Hsu (2025) [26]	Retrospective, cross‐sectional involving people with T2D	EfficientNet‐B3 and EfficientNetV2‐S	Retinal images and clinical metadata	Not reported	eGFR	eGFR < 30 mL/min/1.73m^2^

**TABLE 2:** Characteristics of the included studies' data.

No.	Author and publication year	Development data	Population characteristics	Development data size (*N*)	Internal test size (*N*)	Subgroup analysis	External validation data
1	Betzler (2023) [21]	Singapore Integrated Diabetic Retinopathy Program	Mean age~64 years; 51% male, 49% female; multiethnic (Chinese, Malay, Indian).	4853	1213	Yes	Singapore Epidemiology of Eye Diseases Study (SEED, *N* = 1885) Singapore Macroangiopathy and Microvascular Reactivity in Type 2 Diabetes (SMART2D, *N* = 439) Australian Eye and Heart Study (*N* = 460) Northern Ireland Cohort for the Longitudinal Study of Ageing (*N* = 265)
2*	Bhak (2025) [22]	CHA Bundang Medical Centre (2008–2019)	Mean age 46 years; 54.3% male, 45.7% female; exclusively Korean population.	6458	N/A	Yes	Severance Checkup Health Promotion Centre (2013–2020) (*N* = 2567)
3	Meng (2025) [23]	Shanghai Integrated Diabetes Prevention and Care system (development/internal); 10 multi‐ethnic external cohorts from China, Singapore, Malaysia, Australia and the UK	Multi‐ethnic population with diabetes; mean eGFR ranged from ~76 to 96 mL/min/1.73 m^2^ across the developmental and external cohorts; diabetic retinopathy prevalence 5%–66% across cohorts	85 113 (part of 121 578 participants with diabetes; 486 312 images)	36 465	Yes	Ten cohorts, total *N* = 65 406 (CNDCS, NDSP, PUDM, ECHM, CUHK‐STDR, SEED, UKB, MeLODY, NICOLA, AHES)
4	Prayitnaningsih [24]	Panti Nirmala Hospital and Dr. Saiful Anwar General Hospital, Malang, Indonesia (Nov 2024–Jan 2025)	225 participants (132 controls, 38 T2DM without CKD, 55 T2DM with CKD Stage 3); patients with visible diabetic retinopathy explicitly excluded	180 (approx., 80% patient‐level split)	45 (approx., 20% patient‐level split)	N/A	N/A
5	Cho (2025) [25]	Kyung Hee University Medical Center (KHUMC), South Korea (2006–2023)	People with T2D; discovery cohort *n* = 7028, after exclusions (from 16 637 screened); age, sex not reported by CKD status intext.	7028	5‐fold cross‐validation	Yes	UK Biobank (*n* = 1544)
6	Hsu (2025) [26]	China Medical University Hospital, Taiwan (2006–2018)	People with T2Dl 17 442 patients contributing 42 963 clinical visits: age, sex, and ethnicity not reported by DRKD status.	42 963	8520	Yes	N/A

* Note: Bhak's development data set comprised 36 443 individuals but only 6458 of these individuals live with T2D and were used in the analysis. Similarly, 58 284 individuals were in the external validation data set in total, but only 2567 of these individuals live with T2D [22].

### 3.2 Quality of Included Studies

Quality assessment results are displayed in Table 3. For patient selection, five studies had a low risk of bias; one study [24] was rated high risk because patients with visible diabetic retinopathy and poor‐quality images were explicitly excluded prior to enrolment, which may have produced a less representative case‐mix than a typical screening population. In terms of the index test (i.e., the deep learning model used to detect or predict the presence of DRKD), one study [21] had a high risk of bias, and one study [24] was rated unclear given a small internal test set and a model‐selection process (ablation study) conducted on the same data later used for evaluation. The flow and timing risk of bias was low for most studies, though rated unclear for two studies [23, 24] where the interval between retinal imaging and the reference standard measurement was not explicitly reported. All studies had a low risk of bias regarding the reference standard because eGFR or UACR was used as the reference. Regarding applicability concerns, most studies had a low risk of bias; one study [24] was rated high concern for patient selection given the exclusion of diabetic retinopathy cases and restriction to a single CKD stage, both of which limit generalizability to a typical DRKD screening population. A supplementary PROBAST+AI assessment was also conducted for each included study and is provided in Appendix S3. Four studies [21, 22, 23, 26] demonstrated low overall concern for quality and risk of bias across all domains. One study [25], using a longitudinal risk prediction design, was rated as unclear, reflecting the multimodal ensemble's complexity and the substantial drop in performance observed on external validation. One study [24] was rated as high concern, primarily due to its single‐center design, very small sample size in the disease positive class (*n* = 55), absence of external validation and exclusion of diabetic retinopathy cases limiting representativeness.

**TABLE 3:** QUADAS‐2 results.

Study	Risk of bias				Applicability concerns		
	Patient selection	Index test	Reference standard	Flow and timing	Patient selection	Index test	Reference standard
Betzler (2023) [21]	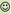	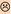	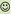	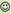	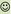	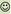	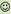
Bhak (2025) [22]	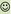	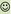	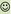	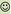	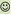	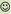	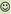
Meng (2025) [23]	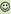	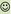	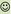	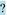	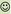	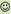	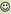
Prayitnaningsih (2026) [24]	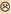	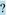	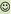	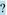	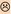	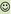	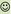
Cho (2025) [25]	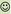	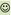	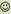	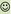	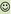	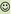	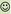
Hsu (2025) [26]	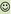	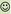	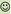	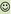	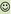	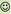	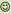

*Note:* 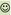 Low risk, 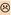 High risk, 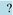 Unclear risk.

### 3.3 Deep Learning Models Performance in Detecting and Predicting the Risk of DRKD

#### 3.3.1 Internal Validation Narrative Review

Some heterogeneity was observed across the included studies, particularly in how DRKD was defined and in the types of deep learning models applied. Three studies [21, 22, 23] defined DRKD using eGFR < 60 mL/min/1.73 m^2^, but with notable differences: one required confirmation on ≥ 2 consecutive visits over 3 months to 2 years, another required a ≥ 25% decline from baseline at follow‐up, and one excluded other causes of kidney injury. One of these [23], additionally accepted UACR ≥ 30 mg/g as an alternative qualifying criterion, an important marker of early kidney damage. One study [24] used a considerably narrower definition, restricted to CKD Stage 3 (eGFR 30–59 mL/min/1.73 m^2^). One study [25], using a longitudinal, risk prediction design, defined incident DRKD using ICD‐9/ICD‐10 diagnostic codes and/or eGFR < 60 mL/min1.73m^2^. eGFR < 60 mL/min/1.73m^2^ was selected as the primary reference criterion for DRKD given its widespread use across the included studies and its consistent availability in routine clinical databases. UACR, while an important early marker of kidney damage that precedes eGFR decline, was inconsistently recorded across the included studies and was therefore incorporated as an additional reference criterion only where reported. The use of different definitions of DRKD and varied modeling approaches across studies means that their results are not directly comparable. Because of these differences, pooling all studies into one summary estimate was not feasible. Instead, we narratively synthesized the findings and performed a focused meta‐analysis on the cross‐sectional studies with comparable definitions.

Betzler et al. [21] used a cross‐sectional dataset from the Singapore Diabetic Retinopathy Programme (SiDRP). They evaluated three models: a retinal image only model, a clinical data only model, and a hybrid model combining retinal images with clinical data, all based on a ResNet18 architecture. On internal validation, the retinal image only model achieved AUC 0.83, sensitivity 0.76, and specificity 0.75; the clinical data only model achieved AUC 0.85, sensitivity 0.78, and specificity 0.76; and the hybrid model an AUC of 0.87, sensitivity 0.79, and specificity 0.77.

Bhak et al. [22] developed a multimodal deep learning model integrating retinal images with urine dipstick data, using a large multi‐center dataset from Korea. On internal validation, the model achieved AUC 0.94, sensitivity 0.86 and specificity 0.88, outperforming both the retinal image‐only model (AUC 0.90) and the urine‐only logistic regression model (AUC 0.88).

Meng et al. [23] analyzed data from 121 578 people with T2D across the Shanghai Integrated Diabetes Prevention and Care system, with external validation across 10 multiethnic cohorts from China, Singapore, Malaysia, Australia, and the United Kingdom (65 406 participants), defining DRKD by either eGFR < 60 mL/min/1.73 m^2^ or ACR ≥ 30 mg/g. Using a ResNet‐50 backbone with weakly supervised momentum contrastive pretraining, they developed a retinal image only model (AUC 0.84), a clinical metadata model (AUC 0.68), and a combined hybrid model (AUC 0.85) on internal validation. Across the 10 external validation cohorts, the retinal image only model achieved AUCs ranging from 0.79 to 0.83.

Prayitnaningsih et al. [24] developed a deep learning model in a single‐center Indonesian cohort of 225 participants, comprising healthy controls, people with T2DM without CKD, and people with T2DM with CKD Stage 3 (*n* = 55). Among several architectures compared (ResNet18, ResNet34, ConvNext, and EfficientNet‐B2), EfficientNet‐B2 performed best, achieving an overall AUC of 0.96, sensitivity of 0.82, and specificity of 0.94 on internal validation. No external validation was undertaken. Because this study used a three‐class outcome in a markedly smaller cohort, rather than the binary eGFR < 60 outcome used by the pooled detection studies, it was not included in the pooled meta‐analysis and is reported narratively.

Hsu et al. [26] analyzed 42 963 clinical visits from 17 442 patients at China Medical University Hospital, collected over a 12‐year period (2006–2018). They compared three model configurations: a single fundus image, a single image combined with age and sex, and bilateral fundus images, using two CNN architectures (EfficientNet‐B3 and EfficientNetV2‐S), with EfficientNet‐B3 consistently outperforming EfficientNetV2‐S across all configurations. They defined DRKD using a considerably more severe threshold of eGFR < 30 mL/min/1.73 m^2^ (advanced CKD Stages 4–5), rather than the broader eGFR < 60 mL/min/1.73 m^2^ threshold used by the other cross‐sectional studies in this review. On a held‐out test set of 8520 visits (573 DRKD‐positive), the best‐performing configuration (bilateral images, EfficientNet‐B3) achieved AUC 0.868, while the hybrid configuration (single image plus age/sex) achieved AUC 0.862 (95% CI: 0.849–0.875), sensitivity 0.796, and specificity 0.756 on internal validation. Because this case definition is not comparable with the eGFR < 60 threshold used by the other cross‐sectional detection studies in this review, this study was not included in the pooled meta‐analysis and is reported narratively.

Out of the six studies in this review, one was a longitudinal study design [25], predicting the 5‐year risk of DRKD using an ensemble of a convolutional neural network (VGG16) trained directly on whole fundus images and a separate deep neural network trained on routine clinical metadata. In a South Korean discovery cohort, the model achieved AUC 0.808, balanced accuracy 0.736, sensitivity 0.704, and specificity 0.768 on internal validation. On external validation in the UK Biobank, performance was lower (AUC 0.722, balanced accuracy 0.635, sensitivity 0.415, specificity 0.856). This study was not included in the pooled meta‐analysis, which was restricted to cross‐sectional detection studies.

To facilitate comparison across all included studies, internal validation performance metrics for retinal image only, clinical data only, and hybrid deep learning models are summarized in Table 4. As shown, hybrid models generally demonstrated superior performance, with AUCs ranging from 0.85 to 0.94, sensitivities between 0.79 and 0.88, and specificities between 0.66 and 0.88.

**TABLE 4:** Performance of retinal image only, clinical data only, and hybrid deep learning models in internal and external validation.

Study	Dataset	Validation type	Type of model	AUC	Sensitivity	Specificity
Betzler (2023) [21]	SiDRP	Internal	Retinal image	0.83 (0.82–0.83)	0.76	0.75
			Clinical data	0.85 (0.84–0.85)	0.78	0.76
			Hybrid	0.87 (0.86–0.87)	0.79	0.77
	SEED	External	Retinal image	0.76 (0.74–0.78)	0.70	0.71
			Clinical data	0.80 (0.78–0.82)	0.79	0.67
			Hybrid	0.83 (0.81–0.85)	0.76	0.74
	SMART2D	External	Retinal image	0.73 (0.69–0.77)	0.64	0.71
			Clinical data	0.70 (0.66–0.77)	0.75	0.61
			Hybrid	0.76 (0.72–0.80)	0.71	0.72
	AHES	External	Retinal image	0.67 (0.61–0.73)	—	—
			Clinical data	0.69 (0.63–0.75)	—	—
			Hybrid	0.70 (0.64–0.75)	—	—
	NICOLA	External	Retinal image	0.64 (0.56–0.71)	—	—
			Clinical data	0.72 (0.65–0.79)	—	—
			Hybrid	0.71 (0.64–0.78)	—	—
Bhak (2025) [22]	CHA	Internal	Retinal image	0.90	—	—
			Urine dipstick	0.93	—	—
			Hybrid	0.94	0.86 (0.81–0.91)	0.88 (0.81–0.91)
	SCHPC	External	Retinal image	0.77	—	—
			Clinical data	0.90	—	—
			Hybrid	0.88	—	—
Meng (2025) [23]	Shanghai	Internal	Retinal image	0.84 (0.84–0.85)		
			Clinical data	0.68 (0.67–0.69)		
			Hybrid	0.85 (0.84–0.85)		
	Ten external cohorts	External	Retinal image	0.79–0.826		
Prayitnaningsih (2026) [24]	Single‐center, Indonesia	Internal	Retinal image	0.96	0.82	0.94
Cho (2025) [25]	KHUMC	Internal	Retinal image	0.61	0.56	0.58
			Clinical data	0.79	0.73	0.71
			Hybrid	0.81	0.70	0.77
	UK Biobank	External	Hybrid	0.72	0.42	0.86
Hsu (2025) [26]	China Medical University Hospital	Internal	Single image	0.86 (0.84–0.87)	0.79	0.77
			Bilateral images	0.87 (0.85–0.88)	0.79	0.79
			Hybrid	0.86 (0.85–0.88)	0.80	0.76

##### 3.3.1.1 Meta‐Analysis of Cross‐Sectional Hybrid Deep Learning Models

To provide an overview of comparable studies, a simple random‐effects meta‐analysis restricted to the three cross‐sectional studies employing hybrid models was conducted. The pooled internal validation metrics were AUC 0.88 (95% CI: 0.86–0.90), sensitivity 0.80 (95% CI: 0.75–0.84), and specificity 0.81 (95% CI: 0.74–0.87) (see Supporting Information: Figures A1–A3; Appendix S2). A large proportion of the observed variability was attributable to between‐study heterogeneity, with *I* ^2^ values of 98.4% for AUC, 93.7% for sensitivity, and 99.8% for specificity (all Cochran's *Q* test *p* < 0.0001). However, the small number of included studies (*n* = 3) limits the reliability of these heterogeneity estimates. Consequently, formal subgroup analyses and meta‐regression were not undertaken (see Strengths and Limitations).

#### 3.3.2 External Validation Review

Four studies [21, 22, 23, 25] in this review conducted external validation. One cross‐sectional study [21] detected the presence of DRKD (Stages G1–G5) using three models: retinal image only, clinical data only, and a hybrid deep learning model. These deep learning models were externally tested in the Singapore Epidemiology of Eye Diseases (SEED) and Singapore Macroangiopathy and Microvascular Reactivity in Type 2 Diabetes (SMART2D) datasets. In the SEED dataset, the retinal image only, clinical data only, and hybrid models achieved AUCs of 0.76, 0.80, and 0.83, with sensitivities of 0.70, 0.79, and 0.76, and specificities of 0.71, 0.67, and 0.74, respectively. In the SMART2D dataset, the corresponding AUCs were 0.73, 0.70, and 0.76, with sensitivities of 0.64, 0.75, and 0.71, and specificities of 0.71, 0.61, and 0.72. These values are lower than those observed in the internal validation datasets, underscoring the importance of external validation and highlighting the need for model calibration. The hybrid deep learning model outperformed the others (see Table 4). A high‐risk coronary artery disease group from the Australian Eye and Heart Study (AHES) and an older adult cohort from the Northern Ireland NICOLA study were used as datasets for supplementary analysis. In the AHES dataset, the retinal image only, clinical data only, and hybrid models achieved AUCs of 0.67 (95% CI: 0.61–0.73), 0.69 (95% CI: 0.63–0.75), and 0.70 (95% CI: 0.64–0.75), respectively. In the NICOLA dataset, the corresponding AUCs were 0.64 (95% CI: 0.56–0.71), 0.72 (95% CI: 0.65–0.79), and 0.71 (95% CI: 0.64–0.78), respectively. Across both cohorts, the hybrid model performed as well as or slightly better than the clinical data only model, and consistently outperformed the retinal image only model, though the absolute gains were modest.

The second study [22] developed three models: retinal image only, a urine dipstick regression model, and a hybrid model to detect DRKD (eGFR < 60 mL/min/1.73m^2^). External validation was performed on the Severance Checkup Health Promotion Centre dataset, where the retinal image only, urine dipstick, and hybrid models achieved AUCs of 0.77, 0.90, and 0.88, respectively. Sensitivity and specificity were not separately reported for this dataset (see Table 4).

The third study [23] to conduct external validation tested its retinal image only model across 10 multi‐ethnic cohorts spanning China, Singapore, Malaysia, Australia, and the United Kingdom, achieving AUCs ranging from 0.79 to 0.83, a notably smaller and more consistent drop from internal validation (AUC 0.84) than observed in the other two studies, likely reflecting the much larger and more diverse development population.

A fourth study [25], using a longitudinal risk prediction design, conducted external validation in the UK Biobank. Performance was lower than on internal validation (AUC 0.72 vs. 0.81), with balanced accuracy 0.64, sensitivity 0.42, and specificity 0.86, illustrating the challenge of generalizing risk prediction models for DRKD across different populations and healthcare systems.

## 4 Discussion

To the best of our knowledge, this is the first systematic review and meta‐analysis to exclusively determine the effectiveness of deep learning models for detecting and predicting DRKD. A limited number of studies met the eligibility criteria for this review. A key finding was that the clinical data, retinal image, or hybrid deep learning models could detect and predict the presence of DRKD, with hybrid models performing best. However, the number of studies (*n* = 6) remained small, sample sizes varied enormously (from 225 to over 121 000 participants), and most studies did not assess ethnicity or sex differences, which impacts the generalizability of these findings. Moreover, only four studies performed external validation, where the performance was reduced, which limits the broader applicability of these findings.

### 4.1 Performance of Retinal Image, Clinical Data and the Hybrid Model

Findings from the meta‐analysis of hybrid models (*n* = 3 studies) demonstrated moderate internal validation performance (pooled AUC 0.88), with sensitivity and specificity of 0.80 and 0.81, respectively (see Appendix S2). While direct statistical comparison with clinical data only or retinal image only models was not possible within this meta‐analysis, results from individual studies consistently showed that hybrid models outperformed single‐modality approaches. This finding is consistent with a study that used retinal images together with clinical data to predict cardiovascular disease [27]. Importantly, in the included studies, hybrid models did not simply append retinal images to an established clinical risk score; rather, they used clinical variables (ranging from 8 to 15 across studies) together with retinal image features within a single deep learning model. This differs from traditional risk prediction equations, which typically include a fixed set of around nine variables (e.g., age, sex, race/ethnicity, eGFR, history of cardiovascular disease, ever smoker, hypertension, BMI, and albuminuria concentration) [28]. Despite the relatively smaller number of clinical variables used in the traditional risk equations, the performance was similar to that of the deep learning models. For example, a study using the traditional risk equation to predict the 5 year risk of developing DRKD from the Chronic Kidney Disease Prognosis Consortium, which included data from 5 222 711 individuals from 28 countries obtained a median C statistic 0.80 (interquartile range, 0.75–0.82) [28] which is similar to the deep learning model with a longitudinal study design [25] which had an AUC of 0.81 for predicting the 5 year risk of DRKD on internal validation. This suggests that while hybrid deep learning models are promising, their incremental benefits over traditional approaches remain modest. Rather than simply continuing to validate existing models, future work should focus on refining model architectures and input data selection to maximize the unique non‐invasive value of retinal imaging. Integrating retinal images with a small number of easily obtainable clinical data (such as diabetes duration) may offer a practical hybrid model that reduces reliance on invasive tests while maintaining or improving predictive accuracy.

Findings from this systematic review and previous research highlight the potential of deep learning for detecting and predicting DRKD from retinal images alone. Particularly in low‐income to middle‐income countries, where health professionals such as ophthalmologists and nephrologists are scarce and access to clinical data may be limited. In India for instance, retinal images are being captured using hand‐held non‐mydriatic cameras [29]. It is important to note that deep learning approaches are likely to augment rather than replace current screening or risk equation methods. For example, the Singapore Eye Lesion Analyzer is currently being deployed as part of the Singapore Integrated Diabetic Retinopathy Programme, where it serves as an initial triage tool for the detection of diabetic retinopathy, glaucoma, and age‐related macular degeneration [30]. In this setting, AI systems provide rapid, automated screening, flagging high‐risk patients who can then be referred for confirmatory assessments and management by ophthalmologists. This augmentation reduces clinician workload, improves screening efficiency, and ensures that specialist attention is directed toward the patients most likely to benefit, while also supporting positive economic outcomes through better resource allocation, rather than attempting to replace clinical decision‐making [31].

### 4.2 Prediction Versus Detection, and the Value of Whole‐Image Deep Learning

Beyond detecting existing disease, predicting the future risk of DRKD carries distinct clinical value, offering a window for early intervention before irreversible kidney damage occurs. Cho et al. [25] is, to our knowledge, the only study in this review to predict incident DRKD directly from retinal images, and its performance, broadly comparable to established risk equations, suggests this is a feasible complement to existing prediction tools. Two further studies suggest the retina holds more signal than has yet been tapped. Shi et al. [32] and Sabanayagam et al. [33] used traditional machine learning built on pre‐specified retinal architecture and vascular parameters, rather than the whole image, achieving AUCs of 0.91 and 0.85 respectively. If hand‐selected features alone can perform this well, a model trained directly on the whole image, unconstrained by which features a human chose to measure, could plausibly capture even more of the kidney‐disease signal the retina holds.

### 4.3 Meta‐Analysis

The meta‐analysis demonstrated that the deep learning models had moderate performance metrics for detecting the presence of DRKD. However, sample sizes across the pooled studies varied enormously, from 4853 to 121 578. Previous research has highlighted that smaller datasets can result in model overfitting, bias, and high variance [13]. Moreover, the populations for training the deep learning models were restricted to Korean, Chinese, Malays, and Indians, which may limit the generalizability of the models to other ethnic groups such as Africans and Caucasians. The meta‐analysis revealed considerable heterogeneity in the pooled estimates of performance metrics (AUC, sensitivity, and specificity), which may be explained by several factors. First, the reference definition of DRKD differed among the studies: Betzler et al. [21] required eGFR < 60 mL/min/1.73 m^2^ confirmed on two consecutive visits, Bhak et al. [22] applied a single eGFR < 60 threshold, and Meng et al. [23] additionally incorporated ACR ≥ 30 mg/g, meaning the pooled models were not all predicting an identical outcome. Second, the studies used markedly different architectures and input features (a ResNet18 convolutional network in Betzler et al. [21], a gradient‐boosted multimodal model incorporating urine dipstick data in Bhak et al. [22] and a ResNet‐50 backbone with weakly supervised contrastive pretraining in Meng et al. [23]), so the underlying predictive tasks were not directly comparable. Third, the development populations differed substantially, a multiethnic Singaporean population, a Korean health‐screening population, and a very large Chinese screening population, introducing variation in DRKD prevalence, retinal pigmentation [34], and imaging practice (including whether pupils were dilated) that may influence performance.

### 4.4 Validation of the Deep Learning Models

Of the six studies in this review, four studies [21, 22, 23, 25] undertook external validation. The first study [21] conducted external validation on four different datasets (Table 2), the second study [22] performed external validation on only one dataset (Table 2), and the third study [23] conducted external validation across ten multi‐ethnic cohorts (Table 2), the most extensive external validation of any included study. The fourth study to conduct external validation was evaluated in the UK Biobank (Table 2). Across all external validation cohorts, hybrid models consistently showed incremental improvements over retinal only. For Bhak et al. [22], hybrid reached 0.88 versus 0.77 retinal only (SCHPC). However, the hybrid advantage over clinical data only models was not consistent externally, clinical only models matched or exceeded hybrid in the NICOLA cohort (0.72 vs. 0.71) and in Bhak et al. [22], where the urine‐dipstick model achieved the highest external AUC of any model (0.90 vs. 0.88). This suggests that, once routinely available clinical or urine data are included, the incremental contribution of retinal images to external generalization may be modest. Performance was lower in the AHES and NICOLA cohorts than in the Singaporean SEED and SMART2D cohorts, likely reflecting their greater demographic and clinical dissimilarity from the multiethnic Singaporean training population. Meng et al. [23], in contrast, showed comparatively consistent external performance (AUC 0.79–0.83) across ten highly diverse cohorts, a notably smaller drop from internal validation (AUC 0.84) than seen in Betzler et al. [21] or Bhak et al. [22] likely reflecting the much larger and more diverse development population. Within Betzler et al. [21], the retinal only model approached hybrid performance in detecting established (Stage 3a–5) DRKD but was weaker at identifying its absence. The small number of late‐stage cases (just under 500) across external cohorts may have limited learning, and later‐stage DRKD may be more strongly reflected in the retina than early DRKD. In all three studies [21, 22, 23], external performance fell below internal validation, underscoring the generalization challenges of AI‐based DRKD risk prediction.

Notably, this pattern of reduced external performance contrasts with the only previous systematic review [19] of machine learning for DRKD. Li et al. [19] reported external performance (AUC 0.83) almost identical to internal (0.84), whereas in the present review external performance fell consistently below internal validation across the retinal‐specific studies, albeit to a lesser degree in the largest and most diverse of these (Meng et.al [23]). This divergence may arise because the external cohorts in this review were predominantly European and Australian and therefore demographically distant from their Asian development populations, exposing generalization gaps that a broader, multi‐modality pool may obscure. Our pooled internal AUC (0.88) also exceeded that of Li et al. [19] despite being derived from far fewer studies, likely reflecting our restriction to retinal‐image based models whereas Li et al. [19] pooled across renal pathology images, renal ultrasounds, and color fundus photographs. These cross‐review differences should be interpreted cautiously given how few retinal‐specific studies exist, but they reinforce a signal that retinal‐image models show genuine promise, while their real‐world value will depend on how they are developed and deployed.

### 4.5 Clinical Relevance of the Deep Learning Models

This systematic review of six studies has shown that hybrid deep learning models combining retinal images with clinical data show better performance for detecting and predicting DRKD than models relying on retinal images or clinical data alone. However, the framework of these models remains largely a “black box” with no explicit explanation of the decision processes behind detecting and predicting DRKD. Although the deep learning models demonstrate good performance, this lack of interpretability can affect the practical applicability of these models in real‐world clinical settings. This is consistent with a study that emphasized the need for deep learning models to be explainable to establish trustworthiness before their adoption in clinical practice [35]. While Bhak et al. [22] employed saliency maps to visualize retinal features contributing to DRKD detections, Prayitnaningsih et al. [24], Cho et al. [25], and Hsu et al. [26], applied Grad‐CAM to identify regions driving predictions, only Meng et al. [23] among the cross‐sectional hybrid studies systematically quantified the relative contribution of each modality and clinical predictor to model decisions. AI predictions of DRKD from retinal images produce outputs that clinicians lack the independent tools to validate. The development of interpretable models capable of clearly communicating which retinal features drove a prediction is therefore an essential precondition for safe clinical deployment.

Considering the varying healthcare resource environments in which these models may be deployed, different implementation strategies may be appropriate. In high‐income settings with established diabetic eye screening programs, hybrid models integrating retinal images with routine clinical data represent the most promising approach. In low‐ and middle‐ income countries where nephrologist access is limited, retinal image only models deployable via low‐cost handheld cameras may offer a more practical prescreening tool. The addition of urine dipstick data, as demonstrated by Bhak et.al [22], represents a promising intermediate strategy that enhances performance without requiring invasive blood tests. Ultimately, the clinical utility of these models will depend not only on their technical performance but on their interpretability, generalisability, and fit with the healthcare systems in which they are intended to operate.

### 4.6 Strengths and Limitations of the Study

Strengths of this study include the robust search methodology, which followed the PRISMA checklist. Following this approach ensured that the bias in study selection was minimized. Despite the limited number of studies that met our eligibility criteria, we were able to synthesize the data in a meta‐analysis (from three studies), which complemented our broader narrative review. Several limitations should be considered when interpreting these findings. First, only six studies met the eligibility criteria, with sample sizes for AI development varying enormously (from 225 to over 121 000 participants), increasing the uncertainty of the pooled estimates for the smaller studies. Second, given the small number of studies contributing to the meta‐analysis (*n* = 3), formal subgroup analyses and meta‐regression were not feasible. The minimum number of studies typically considered necessary for reliable subgroup analysis is ten [36]. Third, the primary risk‐of‐bias appraisal used QUADAS‐2, as no validated AI‐specific tool was available at protocol registration; we additionally applied PROBAST+AI post hoc (Appendix S3), although AI‐specific appraisal was not part of the pre‐registered protocol. Fourth, UACR data were incorporated in only one of the six included studies (Meng et al. [23]) and as UACR abnormalities typically precede eGFR decline, reliance on eGFR < 60 mL/min/1.73 m^2^ alone as the primary reference criterion may have missed early‐stage DRKD characterized primarily by albuminuria, potentially underestimating the models' ability to detect early microvascular injury. Fifth, one included study [24] used a narrower outcome definition restricted to CKD Stage 3 only, and explicitly excluded participants with visible diabetic retinopathy, limiting its comparability with the other four studies and its applicability to a typical DRKD screening population. Sixth, five of the six studies developed their models predominantly in Asian populations (China, Singapore, Taiwan, Korea, and Indonesia), limiting generalisability to African, European, and Indigenous populations, a concern only partly offset by Meng et al. [23] additional external validation in Malaysian, Australian, and UK cohorts. Inclusion was also restricted to English‐language reports, introducing potential language and selection bias.

Further limitations relate to the available evidence and our synthesis. Evidence for prediction, as distinct from detection, rested on a single longitudinal study, so conclusions about predictive performance over time remain preliminary. Differential model performance across sex and ethnic subgroups was rarely reported by the included studies and could not be synthesized here, limiting assessment of equity and subgroup generalisability. The included studies also reported discrimination (AUC) far more consistently than calibration, constraining conclusions about the reliability of the predicted risks for clinical decision‐making. Finally, formal subgroup analyses and meta‐regression to quantify sources of heterogeneity were not feasible given the small number of pooled studies (*n* = 3), for the same reason, publication bias could not be formally assessed (e.g., funnel plot or Egger's test) and cannot be excluded, as studies reporting poor model performance are less likely to be published and may inflate pooled estimates.

### 4.7 Implications for Future Research

Although the performance of the deep learning models was promising, the need for training models on datasets that are broader in terms of age, sex, and ethnicity to enhance generalizability is warranted. Additionally, external validation of these models in larger longitudinal datasets is needed. Past research has highlighted that external validation is crucial before deploying deep learning models in real‐world settings [37]. In addition, future work should involve building deep learning models that can reliably predict the early signs of DRKD before it progresses to late stage renal disease and that are validated across the full spectrum of DRKD severity rather than being restricted to a single stage as in Prayitnaningsih et al. [24] (CKD Stage 3 only) or Hsu et al. [26] (CKD Stages 4–5). Future studies should ensure that UACR is also used as a biomarker for DRKD. Finally, a large‐scale prospective cohort study needs to be undertaken to assess the applicability of these deep learning models in a clinical setting.

## 5 Conclusion

This systematic review synthesized evidence of the use of deep learning models for detecting and predicting DRKD from retinal images. Hybrid models that used both retinal images and clinical data performed slightly better than did models using only images or only clinical data. However, because retinal image only models achieved performance that was close to that of hybrid approaches, they may already be sufficient for initial screening tasks, with hybrid models offering only modest additional benefit. Future work should focus on building models with large, diverse datasets to enhance generalizability and robustness, as well as validating these deep learning models in real‐world settings.

## Author Contributions

**Samuel Ato Gyasi Otabir:** conceptualization, methodology, data curation, formal analysis, investigation, writing – original draft, writing – review and editing. **Teketo Kassaw Tegegne:** methodology, data curation, formal analysis, investigation, writing – review and editing. **Paul Nderitu:** methodology, investigation, supervision, writing – review and editing. **Jiangtao Wang:** methodology, investigation, supervision, writing – review and editing. **Jonathan E. Shaw:** conceptualization, methodology, investigation, supervision, writing – review and editing. **David Owens:** conceptualization, writing – review and editing. **Rebecca Thomas:** conceptualization, writing – review and editing. **Robyn Jennifer Tapp:** conceptualization, methodology, formal analysis, supervision, project administration, writing – review and editing. **Ralph Maddison:** conceptualization, methodology, formal analysis, supervision, project administration, writing – review and editing.

## Funding

This research was supported by a Deakin University Postgraduate Research Scholarship and Diabetes UK (grant number 23/0006601). The research was conducted under a cotutelle agreement between Deakin University and Coventry University.

## Ethics Statement

This study used publicly available data and did not involve primary data collection from human participants or animals. The study was however approved by Coventry University Research Ethics Committee (reference P168783).

## Conflicts of Interest

The authors declare no conflicts of interest.

## Supporting information


**Appendix S1:** Search strategy.
**Appendix S2:** Meta analysis of deep learning hybrid models for internal validation in the detection of DRKD.
**Figure A1:** Summary of pooled AUC.
**Figure A2:** Summary of pooled sensitivity.
**Figure A3:** Summary of pooled specificity.
**Appendix S3:**: Quality assessment using PROBAST+AI.

## Data Availability

S.A.G.O. and T.K.T. had full access to all the data in the study and take responsibility for the integrity of the data and accuracy of the data analysis. Also, S.A.G.O. is responsible for data analysis.
