# Supplementary material for: Detection and Risk Prediction of Diabetes Related Kidney Disease Using Retinal Images: A Systematic Review and Meta‐Analysis
Source: J Diabetes. 2026 Oct 19;18(10):e70271. doi: 10.1111/1753-0407.70271 (PMC13678574; doi:10.1111/1753-0407.70271)
Supplement: Supplementary file 1 [file JDB-18-e70271-s001.docx]

**Supplementary Appendices**

*Supplementary Appendix 1. Search Strategy*

***OVID Medline search: 46 papers***

| **Retinal image** | **Deep learning** | **Diabetic kidney disease** |
| --- | --- | --- |
| (retinal image).ti,ab OR (retinal photo).ti,ab OR (retinal scan).ti,ab OR (fundus image).ti,ab OR (fundus photo).ti,ab OR (fundus scan).ti,ab OR retinography.ti,ab OR ((retina* OR fund* OR ocul* OR eye OR ophthalm*) AND (imag* OR scan* OR photo*)).ti,ab OR (retina* adj4 imag*).ti,ab OR (retina* adj4 scan*).ti,ab OR (retina* adj4 photo*).ti,ab OR (fund* adj4 imag*).ti,ab OR (fund* adj4 scan*).ti,ab OR (fund* adj4 photo*).ti,ab OR (ocul* adj4 imag*).ti,ab OR (ocul* adj4 scan*).ti,ab OR (ocul* adj4 photo*).ti,ab OR (eye adj4 imag*).ti,ab OR (eye adj4 scan*).ti,ab OR (eye adj4 photo*).ti,ab OR (ophthalm* adj4 imag*).ti,ab OR (ophthalm* adj4 scan*).ti,ab OR (ophthalm* adj4 photo*).ti,ab | Deep Learning/ OR Artificial Intelligence/ OR Machine Learning/ OR Neural Networks, Computer/ OR (AI algorithm*).ti,ab OR (DL algorithm*).ti,ab OR (AI model*).ti,ab OR (DL model*).ti,ab OR (Convolutional neural network) .ti,ab OR (Deep neural network).ti,ab OR (Computer neural network).ti,ab | Diabetic Nephropathies/ OR Kidney Diseases/ OR (diabet* nephr*).ti,ab OR (diabet* kidney disease*).ti,ab OR (diabet* adj4 (kidney OR renal OR nephr*)).ti,ab OR (chronic kidney disease* OR CKD).ti,ab OR (renal adj4 (impairment OR disease* OR failure OR insufficienc*)).ti,ab OR (kidney adj4 (disease* OR failure)).ti,ab |

***Embase Logic Grid search: 157 papers***

| **Retinal image** | **Deep learning** | **Diabetic kidney disease** |
| --- | --- | --- |
| 'retina image'/exp OR 'retinography'/exp OR 'retinal image':ti,ab OR 'retinal photo':ti,ab OR 'retinal scan':ti,ab OR 'fundus image':ti,ab OR 'fundus photo':ti,ab OR 'fundus scan':ti,ab OR (retina*:ti,ab OR fund*:ti,ab OR ocul*:ti,ab OR eye:ti,ab OR ophthalm*:ti,ab) AND (imag*:ti,ab OR scan*:ti,ab OR photo*:ti,ab) OR ((retina* NEAR/4 imag*):ti,ab) OR ((retina* NEAR/4 scan*):ti,ab) OR ((retina* NEAR/4 photo*):ti,ab) ((fund* NEAR/4 imag*):ti,ab) OR ((fund* NEAR/4 scan*):ti,ab) OR ((fund* NEAR/4 photo*):ti,ab) OR ((ocul* NEAR/4 imag*):ti,ab) OR ((ocul* NEAR/4 scan*):ti,ab) OR ((ocul* NEAR/4 photo*):ti,ab) OR ((ophthalm* NEAR/4 imag*):ti,ab) OR ((ophthalm* NEAR/4 scan*):ti,ab) OR ((ophthalm* NEAR/4 photo*):ti,ab) OR ((eye NEAR/4 imag*):ti,ab) OR ((eye NEAR/4 scan*):ti,ab) OR ((eye NEAR/4 photo*):ti,ab) | 'deep learning'/exp OR 'artificial intelligence'/exp OR 'machine learning'/exp OR 'convolutional neural network'/exp OR 'deep neural network'/exp OR 'ai algorithm*':ti,ab OR 'dl algorithm*':ti,ab OR 'ai model*':ti,ab OR 'dl model*':ti,ab | 'diabetic nephropathy'/exp OR 'kidney disease'/exp OR 'chronic kidney failure'/exp OR 'diabet* nephr*':ti,ab OR 'diabet* kidney disease*':ti,ab OR ((diabet* NEAR/4 (kidney OR renal OR nephr*)):ti,ab) OR ((renal NEAR/4 (impairment OR disease* OR failure OR insufficienc*)):ti,ab) OR ((kidney NEAR/4 (disease* OR failure)):ti,ab) |

***CINAHL search: 30 papers***

| **Retinal image** | **Deep learning** | **Diabetic kidney disease** |
| --- | --- | --- |
| retina imaging OR retinography OR 'retinal image' OR 'retinal photo' OR 'retinal scan' OR 'fundus image' OR 'fundus photo' OR 'fundus scan' OR (retina* OR fund* OR ocul* OR eye OR ophthalm*) AND (imag* OR scan* OR photo*) OR (retina* n4 imag*) OR (retina* n4 scan*) OR (retina* n4 photo*) (fund* n4 imag*) OR (fund* n4 scan*) OR (fund* n4 photo*) OR (ocul* n4 imag*) OR (ocul* n4 scan*) OR (ocul* n4 photo*) OR (ophthalm* n4 imag*) OR (ophthalm* n4 scan*) OR (ophthalm* n4 photo*) OR (eye n4 imag*) OR (eye n4 scan*) OR (eye n4 photo*) | machine learning OR artificial intelligence OR deep learning OR neural network OR convolutional neural network OR cnn OR deep neural network OR 'ai algorithm*' OR 'dl algorithm*' OR 'ai model*' OR 'dl model*' | diabetic nephropathy OR diabetic kidney disease OR dkd OR diabetic nephropathies OR kidney disease OR renal disease OR renal failure OR kidney failure OR chronic kidney disease OR chronic renal failure OR ckd OR renal insufficiency OR 'diabet* nephr*' OR 'diabet* kidney disease*' OR (diabet* n4 (kidney OR renal OR nephr*)) |

***Cochrane search: 2 papers***

| **Retinal image** | **Deep learning** | **Diabetic kidney disease** |
| --- | --- | --- |
| ("retinal image" OR "retinal photo" OR "retinal scan" OR "fundus image" OR "fundus photo" OR "fundus scan" OR retinography):ti,ab,kw OR ((retina* OR fund* OR ocul* OR eye OR ophthalm*) AND (imag* OR scan* OR photo*)):ti,ab,kw OR ((retina* NEAR/4 imag*) OR (retina* NEAR/4 scan*) OR (retina* NEAR/4 photo*)):ti,ab,kw OR ((fund* NEAR/4 imag*) OR (fund* NEAR/4 scan*) OR (fund* NEAR/4 photo*)):ti,ab,kw OR ((ocul* NEAR/4 imag*) OR (ocul* NEAR/4 scan*) OR (ocul* NEAR/4 photo*)):ti,ab,kw OR ((ophthalm* NEAR/4 imag*) OR (ophthalm* NEAR/4 scan*) OR (ophthalm* NEAR/4 photo*)):ti,ab,kw OR ((eye NEAR/4 imag*) OR (eye NEAR/4 scan*) OR (eye NEAR/4 photo*)):ti,ab,kw | MeSH descriptor: [Deep Learning] OR  MeSH descriptor: [Artificial Intelligence] OR MeSH descriptor: [Machine Learning] OR MeSH descriptor: [Neural Networks, Computer] OR ((AI NEXT algorithm*) OR (DL NEXT algorithm*) OR (AI NEXT model*) OR (DL NEXT model*) OR "Convolutional neural network" OR "Deep neural network"):ti,ab,kw | MeSH descriptor: [Diabetic Nephropathies] OR MeSH descriptor: [Kidney Diseases] OR MeSH descriptor: [Renal Insufficiency, Chronic] OR ((diabet* NEXT nephr*)):ti,ab,kw OR ((diabet* NEXT kidney NEXT disease*)):ti,ab,kw OR (diabet* NEAR/4 (kidney OR renal OR nephr*)):ti,ab,kw OR (renal NEAR/4 (impairment OR disease* OR failure OR insufficienc*)):ti,ab,kw OR (kidney NEAR/4 (disease* OR failure)):ti,ab,kw |

***IEEE Xplore search: 103 papers***

| **Retinal image** | **Deep learning** | **Diabetic kidney disease** |
| --- | --- | --- |
| “retinal image” OR “retinal photo” OR “retinal scan” OR “fundus image” OR “fundus photo” OR “fundus scan” OR “retinography” OR “retina* image” OR “fund* image” OR “ocul* image” OR “ocul* scan” OR “ocul* photo” OR “eye image” OR “eye scan” OR “eye photo” OR “ophthalm* image” OR “ophthalm* scan” OR “ophthalm* photo” | “deep learning” OR "artificial intelligence” OR “machine learning” OR “AI algorithm” OR “DL algorithm” OR “AI model” OR “DL model” OR “convolutional neural network” OR “deep neural network” OR “computer neural network” | “diabetic nephropathies” OR “diabetic nephr*” OR “diabetic kidney disease” OR “diabetic renal disease” OR “chronic kidney disease” OR “CKD” OR “kidney disease” OR “renal impairment” OR “renal disease” OR “renal failure” OR “renal insufficiency” OR “kidney failure” |
| ("All Metadata":“retinal image” OR "All Metadata":“retinal photo” OR "All Metadata":“retinal scan” OR "All Metadata":“fundus image” OR "All Metadata":“fundus photo” OR "All Metadata":“fundus scan” OR "All Metadata":“retinography” OR "All Metadata":“retina* image” OR "All Metadata":“fund* image” OR "All Metadata":“ocul* image” OR "All Metadata":“ocul* scan” OR "All Metadata":“ocul* photo” OR "All Metadata":“eye image” OR "All Metadata":“eye scan” OR "All Metadata":“eye photo” OR "All Metadata":“ophthalm* image” OR "All Metadata":“ophthalm* scan” OR "All Metadata":“ophthalm* photo”) AND ("All Metadata":“deep learning” OR "All Metadata":"artificial intelligence” OR "All Metadata":“machine learning” OR "All Metadata":“AI algorithm” OR "All Metadata":“DL algorithm” OR "All Metadata":“AI model” OR "All Metadata":“DL model” OR "All Metadata":“convolutional neural network” OR "All Metadata":“deep neural network” OR "All Metadata":“computer neural network”) AND ("All Metadata":“diabetic nephropathies” OR "All Metadata":“diabetic nephr*” OR "All Metadata":“diabetic kidney disease” OR "All Metadata":“diabetic renal disease” OR "All Metadata":“chronic kidney disease” OR "All Metadata":“CKD” OR "All Metadata":“kidney disease” OR "All Metadata":“renal impairment” OR "All Metadata":“renal disease” OR "All Metadata":“renal failure” OR "All Metadata":“renal insufficiency” OR "All Metadata":“kidney failure”) | | |

***Web of Science search: 143 papers***

| **Retinal image** | **Deep learning** | **Diabetic kidney disease** |
| --- | --- | --- |
| TS=(retina image OR retinography OR 'retinal image' OR 'retinal photo' OR 'retinal scan' OR 'fundus image' OR 'fundus photo' OR 'fundus scan' OR (retina* OR fund* OR ocul* OR eye OR ophthalm*) AND (imag* OR scan* OR photo*) OR (retina* n4 imag*) OR (retina* n4 scan*) OR (retina* n4 photo*) (fund* n4 imag*) OR (fund* n4 scan*) OR (fund* n4 photo*) OR (ocul* n4 imag*) OR (ocul* n4 scan*) OR (ocul* n4 photo*) OR (ophthalm* n4 imag*) OR (ophthalm* n4 scan*) OR (ophthalm* n4 photo*) OR (eye n4 imag*) OR (eye n4 scan*) OR (eye n4 photo*)) | TS=(machine learning OR artificial intelligence OR deep learning OR neural network OR convolutional neural network OR cnn OR deep neural network OR 'ai algorithm*' OR 'dl algorithm*' OR 'ai model*' OR 'dl model*') | TS=(diabetic nephropathy OR diabetic kidney disease OR dkd OR diabetic nephropathies OR kidney disease OR renal disease OR renal failure OR kidney failure OR chronic kidney disease OR chronic renal failure OR ckd OR renal insufficiency OR 'diabet* nephr*' OR 'diabet* kidney disease*' OR (diabet* n4 (kidney OR renal OR nephr*))) |

***Scopus search: 96 papers***

| **Retinal image** | **Deep learning** | **Diabetic kidney disease** |
| --- | --- | --- |
| retinography OR “retinal image” OR “retinal photo” OR “retinal scan” OR “fundus image” OR “fundus photo” OR “fundus scan” OR (retina* OR fund* OR ocul* OR eye OR ophthalm*) AND (imag* OR scan* OR photo*) OR (retina* W/4 imag*) OR (retina* W/4 scan*) OR (retina* W/4 photo*) (fund* W/4 imag*) OR (fund* W/4 scan*) OR (fund* W/4 photo*) OR (ocul* W/4 imag*) OR (ocul* W/4 scan*) OR (ocul* W/4 photo*) OR (ophthalm* W/4 imag*) OR (ophthalm* W/4 scan*) OR (ophthalm* W/4 photo*) OR (eye W/4 imag*) OR (eye W/4 scan*) OR (eye W/4 photo*) | “machine learning” OR “artificial intelligence” OR “deep learning” OR “neural network” OR “convolutional neural network” OR cnn OR “deep neural network” OR “ai algorithm*” OR “dl algorithm*” OR “ai model*” OR “dl model*” | “diabetic nephropathy” OR “diabetic kidney disease” OR dkd OR “diabet* nephr*” OR “diabet* kidney disease*” OR (diabet* W/4 (kidney OR renal OR nephr*)) OR “chronic kidney disease*” OR CKD OR (renal W/4 (impairment OR disease* OR failure OR insufficienc*)) OR (kidney W/4 (disease* OR failure)) |
| TITLE-ABS-KEY ( ( retinography OR "retinal image" OR "retinal photo" OR "retinal scan" OR "fundus image" OR "fundus photo" OR "fundus scan" OR ( retina* OR fund* OR ocul* OR eye OR ophthalm* ) AND ( imag* OR scan* OR photo* ) OR ( retina* W/4 imag* ) OR ( retina* W/4 scan* ) OR ( retina* W/4 photo* ) ( fund* W/4 imag* ) OR ( fund* W/4 scan* ) OR ( fund* W/4 photo* ) OR ( ocul* W/4 imag* ) OR ( ocul* W/4 scan* ) OR ( ocul* W/4 photo* ) OR ( ophthalm* W/4 imag* ) OR ( ophthalm* W/4 scan* ) OR ( ophthalm* W/4 photo* ) OR ( eye W/4 imag* ) OR ( eye W/4 scan* ) OR ( eye W/4 photo* ) ) AND ( "machine learning" OR "artificial intelligence" OR "deep learning" OR "neural network" OR "convolutional neural network" OR cnn OR "deep neural network" OR "ai algorithm*" OR "dl algorithm*" OR "ai model*" OR "dl model*" ) AND ( "diabetic nephropathy" OR "diabetic kidney disease" OR dkd OR "diabet* nephr*" OR "diabet* kidney disease*" OR ( diabet* W/4 ( kidney OR renal OR nephr* ) ) OR "chronic kidney disease*" OR ckd OR ( renal W/4 ( impairment OR disease* OR failure OR insufficienc* ) ) OR ( kidney W/4 ( disease* OR failure ) ) ) ) | | |

***APA PsycInfo search: No papers***

| **Retinal image** | **Deep learning** | **Diabetic kidney disease** |
| --- | --- | --- |
| retina imaging OR retinography OR 'retinal image' OR 'retinal photo' OR 'retinal scan' OR 'fundus image' OR 'fundus photo' OR 'fundus scan' OR (retina* OR fund* OR ocul* OR eye OR ophthalm*) AND (imag* OR scan* OR photo*) OR (retina* n4 imag*) OR (retina* n4 scan*) OR (retina* n4 photo*) (fund* n4 imag*) OR (fund* n4 scan*) OR (fund* n4 photo*) OR (ocul* n4 imag*) OR (ocul* n4 scan*) OR (ocul* n4 photo*) OR (ophthalm* n4 imag*) OR (ophthalm* n4 scan*) OR (ophthalm* n4 photo*) OR (eye n4 imag*) OR (eye n4 scan*) OR (eye n4 photo*) | machine learning OR artificial intelligence OR deep learning OR neural network OR convolutional neural network OR cnn OR deep neural network OR 'ai algorithm*' OR 'dl algorithm*' OR 'ai model*' OR 'dl model*' | diabetic nephropathy OR diabetic kidney disease OR dkd OR diabetic nephropathies OR kidney disease OR renal disease OR renal failure OR kidney failure OR chronic kidney disease OR chronic renal failure OR ckd OR renal insufficiency OR 'diabet* nephr*' OR 'diabet* kidney disease*' OR (diabet* n4 (kidney OR renal OR nephr*)) |

*Supplementary Appendix 2: Meta analysis of deep learning hybrid models for internal validation in the detection of DRKD.*

**Figure A1. Summary of Pooled AUC.**

**Figure A2. Summary of Pooled Sensitivity.**

**Figure A3. Summary of Pooled Specificity**

*Supplementary Appendix 3: Quality Assessment Using PROBAST+AI*

| **Paper** | **Quality of development** | **Risk of bias (evaluation)** | **Applicability** | **Overall** |
| --- | --- | --- | --- | --- |
| Prayitnaningsih et al. 2025 | HIGH | HIGH | HIGH | Careful leakage handling, but small single-site sample, no external validation, no calibration. |
| Meng et al. 2025 | UNCLEAR | UNCLEAR | UNCLEAR | Largest and most externally validated study, but outcome definition shifts across cohorts. |
| Hsu et al. 0225 | UNCLEAR | HIGH | HIGH | Best-in-class leakage control, but single-centre with no external validation and low PPV due to severe imbalance. |
| Cho et al. 2025 | UNCLEAR | UNCLEAR | UNCLEAR | Only paper with 5-year prediction and thorough calibration reporting; leakage-control transparency is the main gap. |
| Bhak et al. 2025 | UNCLEAR | HIGH | HIGH | Genuine external validation at a separate hospital, undercut by an unreported split unit and no calibration for a rare outcome. |
| Betzler et al. 2023 | UNCLEAR | HIGH | HIGH | Broadest external testing of the six papers, but outcome definitions shift between development and external cohorts. |
